# The effect of online multimedia psychoeducational interventions on the resilience and perceived stress of hospitalized patients with COVID-19: a pilot cluster randomized parallel-controlled trial

**DOI:** 10.1186/s12888-021-03085-6

**Published:** 2021-02-11

**Authors:** Maryam Shaygan, Zahra Yazdani, Adib Valibeygi

**Affiliations:** 1 Community Based Psychiatric Care Research Center, Faculty of Nursing and Midwifery, Shiraz University of Medical Sciences, P.O. Box 713451359, Shiraz, Iran; 2 Faculty of Nursing and Midwifery, Shiraz University of Medical Sciences, Shiraz, Iran; 3 Fasa Neuroscience Circle (FNC), Student Research Committee, Fasa University of Medical Sciences, Fasa, Iran

**Keywords:** Online multimedia psychoeducational intervention, Resilience, Perceived stress, COVID-19

## Abstract

**Background:**

There is evidence suggesting that quarantine might have undesirable psychological impacts on the patients. Therefore, it is important to seek for ways to increase the resilience and alleviate the psychological pressure of the patients who are quarantined due to infection with COVID-19. The present study was conducted to assess an online multimedia psychoeducational intervention regarding the feasibility, adherence, patient satisfaction and effectiveness on resilience and perceived stress of patients hospitalized with confirmed COVID-19.

**Method:**

This was a pilot cluster randomized parallel-controlled trial with hospital wards as the units of randomization. Participants in this fully online trial were 50 consecutive patients who were hospitalized in 2 hospitals in Shiraz, after being diagnosed with COVID-19. Before the beginning of the intervention, four inpatient wards inside two of the hospitals were randomly assigned to either intervention or control conditions. All eligible participants in the wards allocated to the intervention condition received online multimedia psychoeducational interventions during the 2 weeks, whilst the patients in the wards allocated to the control condition were offered the opportunity to receive telephone-based psychological counseling if needed. Psychoeducational interventions mainly included cognitive–behavioural techniques, stress management techniques, mindfulness-based stress reduction and positive psychotherapy. The patients were assessed regarding resilience and perceived stress at baseline and after two weeks.

**Results:**

Of 27 patients starting multimedia psychoeducational interventions, 26 (96.29%) completed post-assessments. A high level of adherence (80.76%) and satisfaction (Mean = 29.42; SD = 4.18) with the online multimedia psychoeducational interventions was found. Compared with the control group, the patients who used online multimedia psychoeducational interventions reported greater resilience (Mean_intervention_ = 81.74; Mean_control_ = 72.86; adjusted t (46) = 2.10; *p* = 0.04; CI: 0.39 to 17.38; dppc2 = 0.83) and fewer perceived stress (Mean_interventio*n*_ = 22.15; Mean_control_ = 29.45; adjusted t (46) = 2.66; *p* = 0.01; CI: − 12.81 to − 1.78; dppc2 = − 0.77) after 2 weeks.

**Discussion:**

The findings of the present study provided a successful first attempt at implementing feasible online multimedia psychoeducational interventions to promote resilience and mitigate stress among the patients who were hospitalized due to infection with COVID-19. The present results could help mental health professionals to determine which psychological techniques should be emphasized to promote patients’ resilience in the context of COVID-19 disease.

**Trial registration:**

Iranian Registry of Clinical Trials, IRCT20201001048893N1. Retrospectively registered, 29 Jan 2021.

## Background

There is evidence suggesting that quarantine might have undesirable psychological impacts on the patients [1]. The most prevalent psychological problems that patients develop following quarantine are known to be fear [1], PTSD [2], stress, insomnia, irritability and low mood [3, 4]. Preliminary data from COVID-19 patients also suggest a high prevalence of depression, anxiety, and sleep disturbance among these patients [5, 6]. In their recent study, Dai et al. [6] found that the prevalence of anxiety and depressive symptoms among patients with COVID-19 is 18.6 and 13.4%, respectively.

According to the emotion hypothetical model of psychological crisis intervention in COVID-19 pandemic, lack of psychological coping methods and isolation can lead to widespread anxiety and fear among patients with COVID-19 [7]. Anxiety about the potential exposure of family members to infection and concern about the health of oneself and significant others add to the distress of patients [8–10]. On the other hand, isolation reduces access and support from family, friends and social support systems, which results in worsening the resilience and perceived stress among patients [7, 9]. These psychological and mental health consequences will add to the cost of managing the illness, if left untreated [11]. Therefore, it is important to seek for ways to increase the resilience of the patients who are infected with COVID-19, which may, in turn, alleviate their perceived stress [12].

Resilience is a multidimensional construct that varies with context, culture and time [13, 14]. Although the operationalization of this construct has considerably varied in the literature [15], various empirical findings and models have described resilience as having three important components; i.e., successful adjustment to stress, propensity to experience positive emotions in the face of stressful situations, and optimism [16, 17]. It has been suggested that cognitive interpretations of individuals about a stressful situation and the way they cope with adverse circumstances seem to be associated with their resilience in the face of distressing events [16, 17]. Given that psychological resilience is a crucial factor reflecting positive adaptation despite adversity, it is essential to increase resiliency in hospitalized patients to reduce the psychological consequences of COVID-19.

However, the fast transmission of the coronavirus has restricted any face-to-face psychological interventions for patients who are quarantined at hospitals. In this context, the use of online multimedia education may provide a safe, innovative opportunity to maintain communication with quarantined patients in order to increase their ability to adapt with this adversity.

To our knowledge, there has been no published original research on the effect of multimedia education on resilience and perceived stress of the infected patients with COVID-19 who are quarantined in hospitals. Moreover, type and severity of stressful situations, cultural norms, social support and government policies are known to be the key factors influencing perceived stress and resilience levels in the face of stressful situations [13, 14]. Research is required to identify the psychological interventions that can be used to enhance resilience levels in the face of the COVID-19 pandemic and to determine whether the goals of pandemic resilience training could be accomplished with internet-based multimedia education that could be widely distributed and self-administered. Therefore, the current pilot randomized study aims to investigate an online multimedia psychoeducational intervention regarding the feasibility, adherence, patient satisfaction and effectiveness on resilience and perceived stress of the patients hospitalized with confirmed COVID-19. In order to control the effects of receiving multimedia psychoeducational interventions and for ethical reasons, participants in the control condition were offered the opportunity to receive telephone-based counseling from a psychologist if needed. It was hypothesized that the patients receiving online multimedia psychoeducational interventions would report increased resilience (primary outcome) and consequently decreased perceived stress (secondary outcome) compared with those in the control group.

## Methods

This study has been reported in accordance with the CONSORT statement [18, 19] that offers guidance for the transparent reporting of randomized controlled studies.

### Study design

We chose to use a cluster randomized parallel-controlled trial with hospital wards as the units of randomization (rather than an individual patient). The primary reason for selection of the cluster randomized design was to protect against the ‘contamination’ that could occur in individually randomized trials [18, 20]. The risk of contamination was minimized by the fact that the hospitalized patients in the intervention and control wards (clusters) were not in contact with each other. Ethical approval was obtained from the local Ethics Committee of Shiraz University of Medical Sciences (IR.SUMS.REC.1399.011). The study was also registered in the Iranian Registry of Clinical Trials (IRCT20201001048893N1).

### Participants and settings

The sample included 50 hospitalized patients with COVID-19 recruited from 4 hospital wards at 2 hospitals (two wards in each hospital) in Shiraz, Iran.

As suggested by Campbell et al. [18], the primary eligibility criterion in a cluster trial is often all clusters in a defined geographical area. Accordingly, the present study incorporated the two hospitals that admitted patients with COVID-19 in Shiraz in April 2020. Patients were included in the study, if they met the following criteria: age over 18 years, laboratory-confirmed COVID-19 infection (real-time reverse-transcriptase–polymerase-chain-reaction: RT-PCR), diagnosed with mild-to-moderate (nonpneumonia/ mild pneumonia, blood oxygen saturation > 93% without oxygen support) or severe (dyspnea, respiratory frequency > 30/min, blood oxygen saturation ≤ 93%) COVID-19 [21–23], willingness to take part in the study, being literate, having been hospitalized during the past 48 h, having internet access and having the ability to work with the media. The following exclusion criteria were applied: diagnosed with critical (respiratory failure, septic shock, and/ or multiple organ dysfunction or failure) COVID-19 [21], having a previous experience of quarantine, being unwilling or unable to continue contributing to the study, having a history of psychiatric disorders or taking psychiatric medications, hospital discharge, death or transfer to the ICU. Patients were not excluded from the study if they were referred to convalescent centers (defined as a transitional form of care provided after a hospital stay but before going home to continue their additional and supplementary treatment and care [24].

### Recruitment

**Fig. 1:**
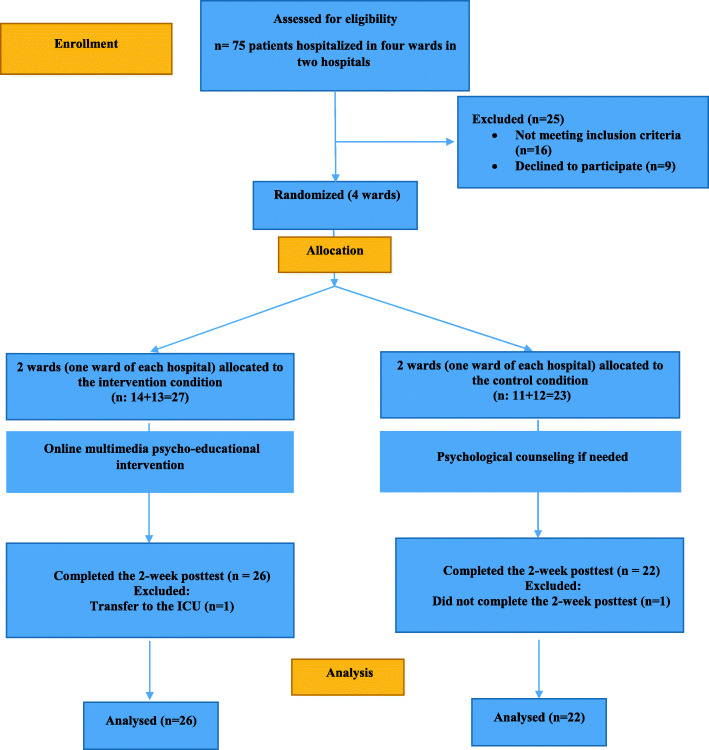
CONSORT Flow Diagram

Patient recruitment was conducted between 1 and 30 April 2020. Two psychologists (not involved in the study) at the study hospitals informed the patients about the purpose of the study and asked them whether they were willing to be contacted by the study staff to undergo screening. The study coordinator contacted the interested patients by telephone and screened the eligible patients until the target number of 50 patients was reached (11–14 patients in each hospital ward). The eligible participants were informed about the voluntary nature of their participation, and online informed consent was obtained from them. Afterwards, online questionnaires were sent to them via WhatsApp (within 48 h of admission to the ward). The patients who did not return the questionnaires were telephoned and encouraged to complete and return the questionnaires. It should be noted that no more than one follow-up attempt was made for each patient. The data were collected anonymously without name lists. Immediately at the end of the second week, the online questionnaires were reapplied and the post-treatment scores were obtained (Fig. 1).

### Randomization

Information about the ‘intervention’ and ‘control’ wards could affect the types of patients recruited (recruitment bias) [25].. Therefore, randomization of wards (clusters) was performed after recruitment of the patients. This was done by an independent observer not involved in this study using a coin toss (allocation concealment). In order to minimize imbalance across the intervention and control groups, a pair-matched randomization of clusters (wards) was used [25]. Accordingly, to ensure comparability of the intervention and control groups, it was attempted to identify the pair of wards in the same hospital, so we randomly allocated one ward in each hospital to the intervention group and the other to the control group. Both hospitals were under the supervision of the Vice Chancellor for Medical Care of Shiraz University of Medical Sciences and provided the same services to all patients with COVID-19.

#### Intervention procedures

##### Online multimedia psychoeducational intervention condition

All eligible patients in the wards allocated to the intervention condition received online multimedia psychoeducational interventions during the 2 weeks. Psychoeducational interventions consisted of 14 daily modules that were based on cognitive–behavioural techniques, stress management techniques, mindfulness-based stress reduction and positive psychotherapy. The patients were asked to complete 1 module per day, which was designed to be 60 min in total. Each module consisted of videos, audio files, educational texts, and one or two exercises related to the module content that were designed by a team of psychologists and psychiatric nurses supervised by the first author. WhatsApp was used to deliver daily multimedia psychoeducational contents (videos, podcasts, and educational texts) to the patients between 9 AM and 9 PM with approximately two-hour intervals.

Cognitive–behavioral modules were designed to teach patients how to recognize and mitigate their cognitive biases, especially in relation to the disease and the likelihood of adverse events due to the disease [26]. In addition, various types of relaxation techniques including progressive muscle relaxation, imagination exercises, and diaphragmatic breathing were taught to the patients via video clips and audio files. Mindfulness techniques were also incorporated to help patients recognize their negative thoughts and emotions about the disease and reduce the intensity and impact of those thoughts and emotions on their stress levels [27]. In this technique, the patients were trained to allow their negative emotions to be there without attempting to alter them or push them away [28]. The patients were encouraged every day to practice these techniques and provide feedback on the techniques that worked best for them and adapted to their conditions. The patients were informed that they were not required to do all the techniques every day. Instead, they were advised to choose the most effective technique for themselves and practice it daily. In order to increase positive emotions and optimism in patients, positive psychotherapy exercises such as “Positive Reminiscence”, “Hope, Optimism, and Posttraumatic Growth”, “Gratitude Text” and “Finding Meaning”, were designed. During the “Positive Reminiscence Exercise”, the patients were encouraged to think about events in the past that evoked positive emotions, visualize the events in detail, and focus on the pleasant feelings arising during the exercise [29]. During “Hope, Optimism, and Posttraumatic Growth” exercise, patients were encouraged to think about the times when important things were lost, but other opportunities transpired. During “Gratitude Text” exercise, the patients were encouraged to write and send a gratitude letter to someone he/she had never properly thanked using WhatsApp [30]. We maintained frequent contact–through text messages and/or phone calls–with the patients to ensure whether or not they have used the modules and applied the techniques appropriately.

### Control condition

All eligible and interested patients in the wards allocated to the control condition were offered the opportunity to receive telephone-based counseling from the psychological team if needed. After the second assessment (T2), the patients in the control condition were offered the multimedia psychoeducational interventions.

### Blinding

The patients were blinded to the patient group assignments and did not know what the other interventions were. In addition, the evaluator and the analyzer of the outcomes were not informed about the patients’ treatment assignments.

### Measures

Data were collected using online questionnaires and forms. Socio-demographic and clinical assessment form developed by the researchers was used to assess the patient’ sociodemographic characteristics (age, gender, marital status and educational level) and clinical features (dyspnoea, fever, cough, fatigue, anorexia, nausea, diarrhea and hemoptysis). The outcome measures were as follows:

### Primary clinical outcome

Resilience was considered as the primary clinical outcome because it was assumed that resilience acts as a protective factor against stress in patients [12]. Resilience was evaluated by the Connor-Davidson resilience scale (CD-RISC) [31]. This scale consists of 25 items rated on a 5-point Likert scale, ranging from 0 (not true at all) to 4 (true nearly all the time). The CD-RISC score could range from 0 to 100, with higher scores reflecting greater resilience. Internal consistency (Cronbach’s alpha) for the full scale is 0.89 [31]. The scale demonstrated good convergent validity, and factor analysis yielded five factors [31]. The Persian version also showed high internal consistency (Cronbach’s alpha = 0.89) and sufficient validity [32, 33].

### Secondary clinical outcome

Perceived stress was estimated employing the Perceived Stress Scale (PSS). The PSS was designed to measure the degree to which situations in one’s life were appraised as stressful [34]. It was a self-report 14-item questionnaire rated on a 5-point Likert scale, ranging from 0 (never) to 4 (very often). Thus, the total score of the scale could range from 0 to 56, with higher scores indicating higher levels of perceived stress. This measure exhibited sufficient reliability (Cronbach’s alpha =0.84–0.86) and validity [34]. The Persian version also showed excellent internal consistency (Cronbach’s alpha = 0.90) and convergent validity [35].

### Feasibility, adherence, and satisfaction with the online multimedia psychoeducational interventions

The feasibility was assessed using the percentage of eligible patients who were enrolled and retained in the study. We defined the study feasible if 70% of patients were adherent to the study [36]. The number of modules and exercises that patients completed (based on self-report) was used as the definition of adherence to the intervention. Full adherence was defined as completing all daily modules and providing feedbacks on daily exercises.

In order to measure the level of satisfaction and to gather the necessary feedback on the online multimedia psychoeducational interventions, the client satisfaction questionnaire adapted to internet-based interventions (CSQ-I) was used [37]. It consists of 8 items answered on a four-point Likert scale ranging from 1 (does not apply to me) to 4 (does totally apply to me). Hence, the total score of the scale varied from 8 to 32. The scale demonstrated excellent internal consistency (McDonald omega = 0.93–0.95) as well as (convergent and discriminant) validity [37]. The Persian version of CSQ-I also demonstrates an excellent internal consistency in the present study (Cronbach’s alpha =0.92). In the present sample, the construct validity of the Persian version of the CSQ-I was confirmed by significant correlations of the CSQ-I score and changes in the scores of resilience (r = 0.41, *P* = 0.03) and perceived stress (r = 0.54, *P* = 0.004) between T1 and T2.

### Statistical analysis

Compliance test for normal distribution was applied using Kolmogorov–Smirnov test. Levene’s test was used to examine the heterogeneity of the variances. Chi-square test was performed to compare the groups concerning demographic and clinical variables. Since the assumptions of analysis of covariance (ANCOVA) were not established [38], independent samples t-tests with adjustment for clustering effect were carried out to evaluate the differences between the two groups with regard to the dependent variables (perceived stress and resilience). Because of the hierarchical structure of the data (with hospital wards as the unit of randomization and patients as the unit of analysis), an adjustment for clustering was needed [39]. To this end, the required Intra-cluster Correlation Coefficients (ICCs) were first calculated by the formula derived by Donner and Klar based on an analysis of variance [40]. Then, the variance inflation factor (VIF) known as the ‘design effect’ was calculated from the ICC. In order to adjust for clustering effect, test statistics based on the t-tests were divided by the square root of the design effect [41]. Between group effect size for the mean differences of groups with unequal sample sizes within a pre-post-control design (dppc2) was calculated according to Morris’s recommendations [42].

Descriptive statistics, such as means, standard deviations (SDs), frequencies and percentages were used to assess the feasibility, adherence, and satisfaction with the online multimedia psychoeducational interventions. Pearson’s correlation coefficients were calculated between the CSQ-I score and changes in resilience and perceived stress scores between T1 and T2. A *p* value < 0.05 was considered to be statistically significant. The analyses were conducted with SPSS® for Windows® version 22.0 (SPSS Inc., Chicago, IL, USA).

### Sample size

Based on the results of a previous study [43], assuming a two-tailed test, α = 0.05, 20% attrition, mean difference = 12, standard deviations (S1 = 12.6, S2 = 9.2), and using the MedCalc software, totally 32 patients were needed to ensure 80% power to detect a significant difference between the intervention and control groups. The calculated sample size was multiplied by the design effect to estimate the effective sample size in the present study (32 × 1.57 = 50). The design effect represents the factor by which the sample size must be increased when a cluster design is used in order to provide the same power as a study with individual allocation and analysis [40]. The design effect for calculating an effective sample size was calculated using the following formula: VIF = 1 + (m-1) ICC, where m was the mean number of individuals per cluster (12.5) and ICC was considered as 0.05 [44].

## Results

Of the 50 eligible patients who started the study, 48 (96%) completed it. Two patients had to be excluded from the study: one patient was excluded because she did not return the post-test questionnaires and the other patient was excluded due to requiring critical care and was transferred to the ICU (Fig. 1). The mean age of the patients was 36.77 years old [standard deviation (SD) = 11.81], and the highest percentage of patients (33.3%) belonged to the age group between 31 to 40 years old (Table 1). The majority of the patients were male (56.2%), married (75%) and about 62.4% of them had primary education (Table 1). The majority of the patients suffered from mild-to-moderate COVID-19 (68.75%), and fatigue was the most common symptom among the patients (43.8%). There were no significant differences between the study groups regarding age group, gender, marital status, educational status, clinical symptoms, severity of disease and length of hospital stay (Table 1).

**Table 1:** Comparison of demographic and clinical variables between control and intervention groups (*n* = 48)

Variables		Group intervention (***n*** = 26)	Control (***n*** = 22)	X^**2**^(df)/ t(df)	***P***-value
**Age group,** ***n*** **(% of total)**	**18–30 years**	8 (16.7%)	7 (14.6%)	3.08 (3)	0.37
	**31–40 years**	11 (22.9%)	5 (10.4%)		
	**41–50**	6 (12.5%)	7 (14.6%)		
	**50 < years**	1 (2.1%)	3 (6.3%)		
**Gender,** ***n*** **(% of total)**	**Male**	13 (27.1%)	14 (29.1%)	0.90 (1)	0.34
	**Female**	13 (27.1%)	8 (16.7%)		
**Marital status,** ***n*** **(% of total)**	**Single**	7 (14.6%)	5 (10.4%)	0.11 (1)	0.73
	**Married**	19 (39.6%)	17 (35.4%)		
**Education,** ***n*** **(% of total)**	**High school or less**	16 (33.3%)	14 (29.1%)	4.11 (3)	0.24
	**Diploma**	2 (4.2%)	5 (10.4%)		
	**Bachelor**	6 (12.5%)	3 (6.3%)		
	**M.Sc./ Ph.D**	2 (4.2%)	0		
**Clinical symptoms,** ***n*** **(% of each group)**	**Dyspnoea**	7 (26.9%)	8 (36.4%)	0.49 (1)	0.48
	**Fever**	11 (42.3%)	7 (31.8%)	1.17 (1)	0.27
	**Cough**	10 (38.5%)	7 (31.8%)	0.67 (1)	0.30
	**Fatigue**	13 (50%)	8 (36.4%)	0.90 (1)	0.34
	**Anorexia**	6 (23.1%)	10 (45.5%)	2.68 (1)	0.10
	**Nausea/Vomiting**	2 (7.7%)	2 (9.1%)	0.03 (1)	0.86
	**Diarrhea**	4 (15.4%)	1 (4.5%)	1.5 (1)	0.22
	**Haemoptysis**	0	2 (9.1%)	2.46 (1)	0.11
**Severity of disease,** ***n*** **(% of each group)**	**Mild-to-Moderate**	19 (73.07%)	14 (53.84%)	0.49 (1)	0.48
	**Severe**	7 (26.92%)	8 (46.15%)		
**Length of hospital stay, Mean (SD)**		9.12 (2.79)	9.59 (2.73)	0.59 (46)	0.55

*SD* standard deviation

**Fig. 2:**
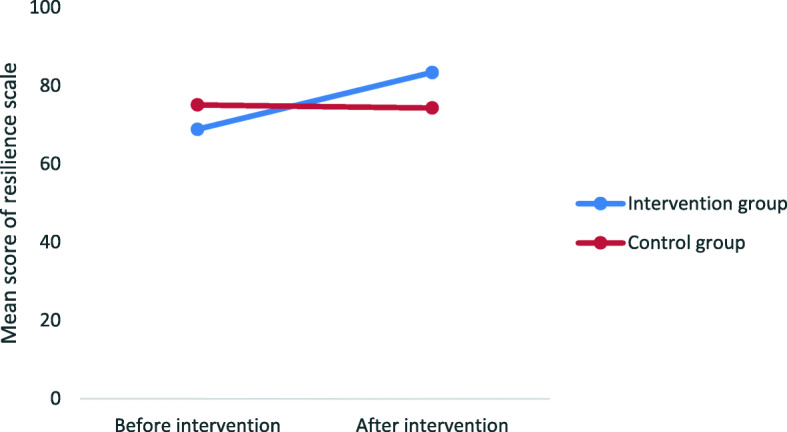
Changes of adjusted mean scores of resilience scale before and after intervention

**Fig. 3:**
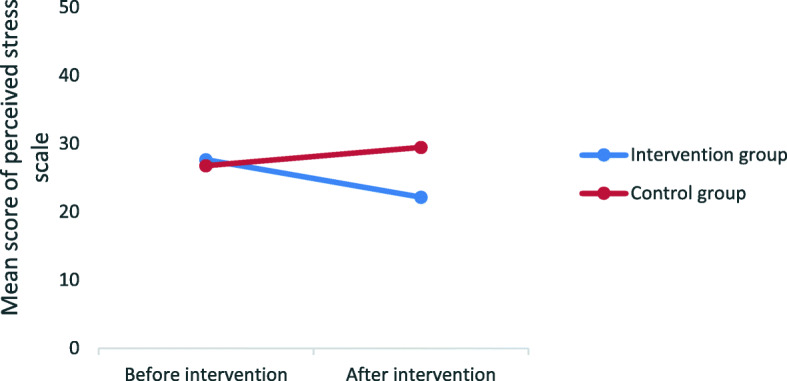
Changes of adjusted mean scores of perceived stress scale before and after intervention

The Kolmogorov-Smirnov test showed the normal distribution of the quantitative variables. Levene’s tests were not significant and, consequently, equal variances were assumed. At baseline, the ICC was found to be 0.004 for the resilience score and 0.0001 for the perceived stress score in the four clusters (the whole baseline sample). Based on the results of the independent samples t-tests with adjustment for the clustering effect, both groups were homogeneous and comparable with respect to their resilience (Mean_intervention_ = 67.49; Mean_control_ = 73.61; adjusted t(46) = 1.19; *P* = 0.23; 95% CI: − 16.42 to 4.18) and perceived stress (Mean_intervention_ = 27.65; Mean_control_ = 26.77; adjusted t(46) = 0.29; *P* = 0.77; 95% CI: − 5.21 to 6.97) scores at baseline. However, immediately after the intervention, there were significant differences between the two groups with regard to resilience (Mean_intervention_ = 81.74; Mean_control_ = 72.86; adjusted t (46) = 2.10; *p* = 0.04; CI: 0.39 to 17.38; dppc2 = 0.83) and perceived stress scores (Mean_intervention_ = 22.15; Mean_control_ = 29.45; adjusted t (46) = 2.66; *p* = 0.01; CI: − 12.81 to − 1.78; dppc2 = − 0.77) (Table 2). The findings suggest that compared with the control group, the intervention group had significantly greater improvements in the scores of resilience and perceived stress after two weeks (Figs. 2 and 3).

**Table 2:** Comparison of resilience and perceived stress scores between control and intervention groups before and after intervention (*n* = 48)

variable	ICC	Time	Intervention group (***n*** = 26)	Control group (***n*** = 22)	T(46) ^**a**^	***P***-value	Effect Size d_**ppc2**_
**Resilience (Mean ± SD)**	0.004	**Before intervention**	67.49 ± 19.12	75.09 ± 16.09	−1.19	0.23	0.83
		**After intervention**	81.74 ± 11.57	74.32 ± 17.83	2.10	0.04	
**Perceived stress (Mean ± SD)**	0.0001	**Before intervention**	27.65 ± 10.12	26.77 ± 10.82	0.29	0.77	−0.77
		**After intervention**	22.15 ± 7.95	29.45 ± 10.96	−2.66	0.01	

*a* Adjusted t-test for clustering

Of 27 patients starting the multimedia psychoeducational interventions, 26 (96.29%) completed post-assessments at T2 (one patient had to be excluded from the study due to transfer to the ICU). Of these, 21 patients (21/26, 80.76%) fully adhered to the interventions by completing all 14 modules and providing feedbacks on all the exercises. In those not fully adhered to the interventions, three patients completed all 14 modules (based on self-report) but did not provide any feedback on the exercises, one patient completed 75% of the modules and one patient completed 50% of the modules.

In the control group, 23 patients started the study and 22 (95.65%) completed the post-assessments at T2. One patient had to be excluded from the study because she did not return the post-test questionnaires. Two patients requested for obtaining telephone-based psychological counseling during the 2 weeks.

The satisfaction with online multimedia psychoeducational interventions ranged from mean 3.50 (SD = 0.81) on item 6 “The training helped me deal with my problems more effectively” to mean 3.80 (SD = 0.49) on item 8 “I would come back to such a training if I were to seek help again”. The average total CSQ-I score was 29.42 (SD = 4.18), with 15 patients (15/26, 57.7%) reporting the highest possible total score. None of the patients received the lowest possible score. The CSQ-I score was significantly correlated to the changes in the scores of resilience (r = 0.41, *p* = 0.03) and perceived stress (r = 0.54, *p* = 0.004) between T1 and T2. This implied that on average, patients with more enhancement of resilience and larger reductions in perceived stress appeared to be more satisfied with the online multimedia psychoeducational interventions.

## Discussion

Contagious diseases outbreaks might lead to irreparable psychological trauma amongst patients and societies, which imposes a heavy financial burden on the healthcare system. The existing evidence highlights that hospitalized patients with COVID-19 suffer from high levels of stress and depression [6, 45]. Without timely psychological interventions, there is a risk that these psychological symptoms will evolve into severe mental disorders such as post-traumatic stress disorder [8]. Therefore, finding ways to relieve this damage can improve mental health and reduce psychological distress at both individual and social levels. The findings of the present study provided a successful first attempt at implementing feasible online multimedia psychoeducational interventions to promote resilience and mitigate stress among the patients who were hospitalized due to infection with COVID-19.

Despite various studies on the psychological health of patients with COVID-19, most studies are limited to a cross-sectional design. Only three randomized controlled trials are available for evaluation of the effects of psychological interventions in patients with COVID-19 (one face to face, one internet-based, and one telephone-based) [40, 46, 47], although none assessed resilience in the patients. Wei and colleagues showed the pleasant effectiveness of face-to-face cognitive behavioral therapy (CBT) in improving the psychological distress among hospitalized patients with COVID-19 [47]. The other randomized controlled trial demonstrated the positive effect of progressive muscle relaxation on anxiety and sleep quality of patients with COVID-19 [40]. Li et al., also found a positive effect of an internet-based integrated intervention on mild to moderate depression and anxiety symptoms in patients with COVID-19 [46]. Consistent with these findings, our results showed that online multimedia psychoeducational interventions targeting cognitive appraisals (specially in relation to the disease), stress management, positive emotions and optimism could significantly promote resilience and mitigate stress levels among the patients who were hospitalized due to infection with COVID-19. It seems that our online multimedia interventions resulted in a rapid improvement on resilience and stress levels, which could be useful in the management of psychological distress in COVID-19 patients.

According to the literature, there are different ways in which the present educational package might influence resilience and stress in patients hospitalized with COVID-19. Resilience has been defined as the ability of an individual to cope positively with adversity [16]. It has been suggested that cognitive appraisals substantially influence how an individual copes with stressful events [48]. Researchers have identified positive appraisals as an important influencing factor in psychological resilience [16, 49]. Therefore, teaching patients to recognize and mitigate their negative appraisals, especially regarding their disease, might be helpful in promoting their resilience in the face of such distressing event. Moreover, enhancing positive emotions and optimism through positive psychotherapy exercises might lead to acceleration of patients’ ability to adjust to the novel coronavirus disease. These findings are in line with those of other studies indicating that positive emotions and optimism facilitate resilience under stressful circumstances [17]. We believe that stress reduction techniques could be helpful in reducing perceived stress as well as in facilitating resilience among patients [12, 17]. In addition to the educational benefits of the program, a sense of availability of resources [16] and connectedness [16, 50] with mental health professionals might contribute to relieving the patients’ stress and elevating their resilience in this tough situation. Receiving daily modules and feeling connected to mental health professionals could provide reassurance to patients hospitalized with COVID-19 that they are not forgotten and that their needs are just as important as those of the patients with non-contagious diseases. This was reflected in the high level of patients’ adherence and satisfaction with the online multimedia interventions.

A key objective in the promotion of mental health is to offer interventions that will be available to everyone. To date, the availability of smartphones and online services has allowed mental health professionals to provide early mental health services on site for those who need mental care [40]. Online psychological interventions can be cost-effective and time-efficient [47]. Therefore, they increasingly complement face-to-face psychological interventions [47]. A growing body of research supports the efficacy of technology-based (i.e., computer/Internet) interventions on resilience, wellbeing, quality of life, optimism, coping strategies, anxiety, stress and depression among university students, dementia carers, burn patients, chronically ill adolescents and patients with cancer [51–55]. The present findings are consistent with those of Parks et al. showing a reduction in anxiety and an increased resilience among participants in a web-based psychological intervention grounded in positive psychology, cognitive–behavioural therapy and mindfulness–based stress reduction [17]. Our study extended previous research in this area, because it investigated the beneficial effects of online multimedia psychoeducational interventions to promote patients’ resilience in the context of contagious diseases, such as the novel coronavirus disease. Given that the fast transmission of the coronavirus between people hinders the traditional face-to-face psychological interventions, online interventions can be efficient ways to implement preventive and therapeutic mental health interventions in COVID-19 patients.

According to the results, the satisfaction scores were on average very high, showing that most patients reported to be satisfied with the delivered online interventions. Patients who had more changes in the scores of resilience and perceived stress were more satisfied with the received interventions. Thus, these findings demonstrate the ability of the satisfaction questionnaire (CSQ-I) to discriminate between more and less satisfied intervention users. However, it has to be noted that most of the CSQ-I items cover the user’s satisfaction with the general quality of the online intervention rather than focusing on specific characteristics of the intervention such as usability and simplicity of the intervention content. It would be valuable if future studies evaluate additional quality dimensions of online psychological interventions that may also be relevant for clinical success.

The majority of the patients adhered to the online multimedia psychoeducational interventions, indicating its applicability. Perceived ease of use and perceived usefulness of a program can determine the attitude and behavioral intention towards use, influencing adherence [56]. Patients who are quarantined due to infection with COVID-19 are experiencing high levels of boredom, loneliness and isolation. We believe that loneliness, willingness to communicate with others, willingness to seek support, having sufficient time, and a high level of satisfaction with interventions may result in a high level of adherence to the delivered online interventions. However, because full adherence was operationalized by the self-reported number of completed modules and exercises, so it would not be of value to identify the correlation between adherence with satisfaction and clinical outcomes. Future research should use multiple measures of adherence, including objective measures, to investigate the correlation between adherence to online psychological interventions and effectiveness in terms of clinical outcomes. Moreover, splitting patients by usage level (eg, time spent on the modules) may provide very interesting additional information. Identifying which subgroup of patients adhere to the online psychological interventions and which factors are associated to these possible differences in adherence will help identify which patients can be targeted and how the online psychological interventions can be improved. Further studies are needed to clarify what patient- and intervention-related factors are determinants of adherence to the online psychological interventions [57]. Such knowledge on adherence level to online psychological interventions will enable more individualized treatment decisions.

We believe that the present findings are significant, as they support implementing feasible online psychological interventions to promote resilience and mitigate stress levels in patients with COVID-19 who had limited access to face-to-face communication and traditional psychological interventions. However, we also acknowledge several limitations. The small sample size is the main limitation of the present study. Therefore, further studies with larger sample sizes are required to confirm the findings of the present study. Another remarkable limitation of this study is the lack of a long-term follow up to identify the stability of the obtained therapeutic benefits. Although due to the fast transmission of the coronavirus,and the fact that families could not visit their hospitalized family members, the quality of family support received by patients might affect the results of our study. Adherence was estimated based upon self-report and not on objective measures. Although self-reported adherence has been shown to correlate with clinical outcomes [58, 59], the use of self-report may overestimate adherence [60]. Therefore, it would be most valuable if future studies included both self-report and objective measures of adherence. Finally, the current study only examined two psychological outcomes; i.e., resilience and perceived stress, while there are many other outcomes that may be affected by online psychoeducational interventions for patients with COVID-19. Hence, further studies are required to shed light on this issue.

## Conclusions

In summary, our findings support the beneficial effects of an online multimedia psychoeducational intervention grounded in cognitive–behavioural therapy, mindfulness–based stress reduction and positive psychotherapy on the resilience and perceived stress of patients hospitalized with confirmed COVID-19. The present results could help mental health professionals to determine which psychological techniques should be emphasized to promote patients’ resilience in the context of COVID-19 disease. Given that the fast transmission of the coronavirus between people hinders the traditional face-to-face psychological interventions, feasible online interventions can be regarded as a cost-effective and convenient tool to protect the patients from the undesirable psychological damages of the quarantine.

## Data Availability

The datasets used and/or analyzed during the current study are available from the corresponding author on reasonable request.

## Abbreviations

COVID-19: Coronavirus Disease 2019
PTSD: Posttraumatic Stress Disorder
CONSORT: Consolidated Standards of Reporting Trials
CD-RISC: Connor-Davidson Resilience Scale
PSS: Perceived Stress Scale
CSQ-I: Client Satisfaction Questionnaire Adapted to Internet-based Interventions
ANCOVA: Analysis of Covariance
ICCs: Intra-cluster Correlation Coefficients
VIF: Variance Inflation Factor
CI: Confidence Interval
SD: Standard Deviation
ICU: Intensive Care Unit
